# Periodontal Diseases and Dental Caries in Children with Type 1 Diabetes Mellitus

**DOI:** 10.1155/2015/379626

**Published:** 2015-08-04

**Authors:** Marta Novotna, Stepan Podzimek, Zdenek Broukal, Erika Lencova, Jana Duskova

**Affiliations:** School of Dental Medicine, First Faculty of Medicine and General University Hospital, Charles University, Karlovo Namesti 32 and Katerinska 32, 121 11 Prague, Czech Republic

## Abstract

Type 1 diabetes mellitus is a chronic metabolic disease of an autoimmune origin with early manifestation predominantly in the childhood. Its incidence has been rising in most European countries. Diabetes has been intensively studied by all branches of medicine. There were a number of studies investigating oral consequences of diabetes; however, unambiguous conclusions were drawn only for the relationship between diabetes and periodontal impairment. Many studies confirmed higher plaque levels and higher incidence of chronic gingivitis both in adults and in children with diabetes. Juvenile periodontitis is rare both in healthy subjects and in those with type 1 diabetes. Yet certain findings from well-conducted studies, for example, differences in oral microflora or the impact of metabolic control of diabetes on periodontal health, indicate a higher risk of periodontitis in children with type 1 diabetes. As for the association of diabetes and dental caries, the results of the studies are inconsistent. However, it was found that some risk factors for dental caries are either more or less prevalent in the diabetic population. Despite an extensive research in this area we have to acknowledge that many questions have remained unanswered. There is a need for continued, thorough research in this area.

## 1. Introduction

### 1.1. Diabetes: Definition and Current Classification

Diabetes mellitus is a common term for a group of chronic metabolic diseases, the basic feature of which is hyperglycemia.

Currently, classification of diabetes by ADA (American Diabetes Association) is being used, which emphasizes etiology of the disease (Table 1) [1].

Previously used classification by WHO (World Health Organization) from 1985 was based on the treatment of the disease and distinguished diabetes mellitus (DM), impaired glucose tolerance (IGT), and gestational diabetes mellitus (GDM). Type 1 diabetes belongs to the subgroup insulin-dependent diabetes mellitus according to this classification [2].

In ICD-10 (International Statistical Classification of Diseases and Related Health Problems, 10th revision), diabetes is classified under codes E10–E14, where type 1 diabetes mellitus is the title of E10 code [3].

This review focuses on the relationship between type 1 diabetes and oral health.

### 1.2. Type 1 Diabetes Mellitus: Etiology

Diabetes mellitus is a serious and relatively common chronic childhood disease. The basis of the disease is an autoimmune insulitis leading to a destruction of the beta cells of the pancreatic islets of Langerhans producing hormone insulin. This results in the clinical manifestation of the disease. The disease manifests itself in genetically predisposed individuals (polygenic genetic predisposition) after interaction of genetic and environmental factors [4]. It is estimated that, in the pathogenesis of the disease, the genetic and nongenetic factors are involved approximately to the same extent. Major environmental factors presumably associated with an increased risk of autoimmune insulitis include enterovirus infections (coxsackievirus type B) [5, 6], some nutritional factors (e.g., early exposure to cow's milk proteins, high intake of cow's milk products in the childhood, duration of breastfeeding, the effect of nitrates and nitrites, and vitamin D deficiency) [7], and perinatal and early childhood factors (e.g., higher age of the mother, lower birth order, and limited contact with other children) [8]. It is probably environmental factors, that is, changes in the exposure to certain nongenetic factors, that are responsible for a dramatic increase in an incidence of type 1 diabetes over the recent decades, because such an increase in the proportion of risk genotypes for type 1 diabetes in the population is not likely. As the pathogenesis of diabetes is still not fully explained, there are no effective preventive measures [9].

The incidence of the disease is predominantly nonfamilial; only 5–10% of the patients are siblings of diabetic children or children of diabetic parents [4]. In monozygotic twins, a simultaneous occurrence of type 1 diabetes was demonstrated in 23–53% of them [9].

The first clinical signs of diabetes in children include polyuria and polydipsia caused by an exceeded renal threshold for glucose excretion, followed by a gradual metabolic breakdown with typical symptoms.

Laboratory findings include hyperglycemia, glycosuria, and ketonuria. In order to evaluate the metabolic control of diabetes and treatment success rate, the blood levels of glycated hemoglobin (HbA1c) are monitored, as they reflect the blood glucose fluctuations over the last six weeks.

The treatment of type 1 diabetes is currently based on an intensified insulin therapy, which should mimic the normal insulin secretion, on regular self-monitoring of specific metabolic biomarkers in the patient, and on an education of the patient and his/her family [4].

### 1.3. Type 1 Diabetes Mellitus: Prevalence in Europe and in the Czech Republic

The incidence of type 1 diabetes differs significantly among diverse ethnic groups and nations. This is due to genetic differences and probably also due to still-not-fully-explained nongenetic factors.

**Figure 1:**
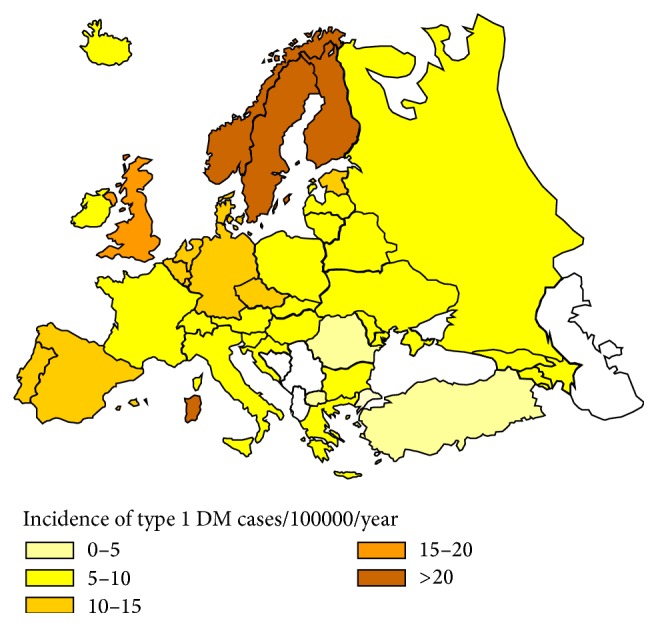
Incidence of type 1 diabetes in children up to 14 years of age/100000/year over the years 1990–1994 [12].

In Europe, there is a north-south gradient in the disease incidence. The highest incidence is observed in Finland (40.2/100000/year), while the lowest incidence rates are reported by Balkan countries, particularly by Macedonia (3.2/100000/year), with the exception of the island of Sardinia (Figure 1) [4, 10–12].

When compared to other European countries, the Czech Republic has an intermediate but steadily rising incidence of diabetes. Over the recent years, type 1 diabetes mellitus was diagnosed in approximately three hundred Czech children per year [9].

## 2. Oral Health in Children with Type 1 Diabetes

The relationship of type 1 diabetes as an underlying disease and different aspects of oral health has been investigated by a number of studies worldwide. Even though some associations have been confirmed, some others are still being discussed and the results of individual studies are often controversial not only due to methodology differences but also due to multifactorial etiology of most oral pathologies. After carrying out a thorough search of PubMed database, the authors can state the following points. Only a limited number of studies have been performed in children with type 1 diabetes probably due to a relative rareness of the disease. Nevertheless, the majority of the results of the research performed in adults with type 1 diabetes could be applied also to the child population. Case studies and reviews of the literature focusing on diabetic adults regardless of diabetes type and treatment are most frequent; however, the applicability of their findings to children with type 1 diabetes is debatable, given significant differences between the two types of diabetes and with respect to a higher proportion of type 2 diabetics in the studies.

### 2.1. Gingivitis, Dental Plaque, Calculus, and Periodontitis

Association between the terms mentioned above could be explained in a simplified way. It is now well accepted that bacteria in dental plaque are the major villains of periodontal diseases, which are infections of the structures around the teeth (the gums, the cementum, the periodontal ligament, and the alveolar bone). Calculus is a mineralized form of dental plaque which facilitates plaque deposition and irritates the gums. In the earliest stage, only the gums are affected (gingivitis). Without treatment, the infection spreads after a period of time and in the end all of the supporting tissues are involved (periodontitis). After bone resorption, the teeth become loose and finally fall out [13].

A vast majority of studies have concluded that the incidence of chronic gingivitis in patients with type 1 diabetes is significantly higher than that in the healthy population and it increases with age. In a group of diabetic children aged 5–9 years, the mean value of gingival inflammation index (score of 0–3) was 1.54 ± 0.5, while in the control group it was 1.14 ± 0.5. In a group of diabetic children aged 10–14 years the mean value of gingival inflammation index versus the control group was 1.98 ± 0.6 and 1.17 ± 0.5, respectively [14]. In a Swiss clinical trial on experimental gingivitis induced by refraining from oral hygiene for three weeks, there were no differences in the plaque index scores or in the composition of bacterial plaque between the type 1 diabetics and healthy controls, but the diabetics responded to plaque irritation by an earlier developed and more severe gingival inflammation, which corresponded to a significantly higher levels of some inflammatory biomarkers in crevicular fluid [15, 16]. Another research performed in a large group of Brazilian child diabetics with a mean age of 13 ± 3.5 years observed gingivitis and periodontitis in 21% and 6% of the study subjects, respectively [17]. In a group of Lithuanian children with diabetes aged 10–15 years, the prevalence of gingivitis versus the control group was 27% and 13%, respectively [18]. Similarly, there are reports of a higher incidence of dental plaque [19] and earlier and heavier formation of calculus in diabetic children. The significant differences between the diabetic and healthy individuals appear in adolescence [14, 18].

Although periodontitis does not belong to clinical manifestations of any type of diabetes mellitus, it is still being labeled as “the sixth chronic complication of diabetes.” It has been confirmed that, in individuals with diabetes, there is about a three times higher risk of periodontitis. Thus, diabetes is considered to be a predisposing factor for periodontitis [20]. In the diabetic patients, the periodontal disease develops at a younger age than in the healthy population [21, 22]. In children with diabetes, the periodontal impairment usually manifests in the adolescence [19, 23] and sometimes even earlier [24]. It was confirmed that there is an association between poorly controlled diabetes (higher HbA1c levels) and a development of periodontitis, even in children with type 1 diabetes [17, 20, 22, 25, 26]. Some studies show relationship between the duration of diabetes and severity of periodontitis [17, 21]. On the other hand, it was confirmed that there is a negative effect of periodontitis on blood glucose levels. This is due to an increased insulin resistance of tissues in reaction to systemic inflammatory mediators [20]. Recently, a presumption that treatment of periodontitis results in an improved metabolic control of diabetes has been confirmed [20], although some earlier studies did not support this hypothesis [25, 27, 28]. According to the recent clinical trials, a successful treatment of periodontitis decreased the HbA1c levels (reflecting a long-term diabetes control) of 0.4% [20], but this was observed mainly in patients with type 2 diabetes [29].

The onset and progression of periodontitis in diabetic patients are probably induced by diabetic microangiopathy, impaired immune response and a lower resistance to infections, different oral microflora, and disorders in collagen metabolism [23].* In vitro* a direct negative effect of hyperglycemia and hypoglycemia on periodontal cells has been demonstrated. Hyperglycemia has also an indirect adverse effect, stimulating immune system cells to release inflammatory cytokines [30]. Recently, a number of studies have been published dealing with biochemical principles of periodontal damage in diabetes.

### 2.2. Impaired Immune Response in Diabetes and Periodontitis

Hyperglycemia caused by diabetes mellitus can alter immune system in many ways. First of all, it increases salivary concentration of glucose as well as its concentration in gingival crevicular fluid. This increased availability of glucose in the environment of oral cavity increases proliferation of periodontopathic and cariogenic bacteria and increases oral inflammation. Presence of elevated levels of proinflammatory mediators in the gingival crevicular fluid of periodontal pockets of poorly controlled diabetics, compared to nondiabetics or well-controlled diabetics, resulting in significant periodontal destruction with an equivalent bacterial challenge has been shown [31–33].

Hyperglycemia caused by diabetes mellitus can lead also to microangiopathy. Endothelial cells lining blood vessels use more glucose than usually and form more glycoproteins on their surface and basement membrane grows thicker and weaker. The vessel walls become thick and weak and vessels bleed easily and leak proteins. These vascular changes in periodontium decrease polymorphonuclear cells functions such as chemotaxis, adherence, phagocytosis and migration, oxygen utilization, and antigens elimination leading to progression of periodontitis.

Hyperglycemia also increases the formation of advanced glycation end-products. The overexposure of proteins (such as collagen) or lipids to aldose sugars induces nonenzymatic glycation and oxidation. These glycosylated products can create complex molecules, reducing collagen solubility and increasing levels of proinflammatory mediators responsible for the degradation of connective tissues. Changes to collagen metabolism result in accelerated degradation of both nonmineralized connective tissue and mineralized bone [32, 34]. The interaction of advanced glycation end-products with target cells, such as macrophages, via cell-surface polypeptide receptors stimulates the production of cytokines and matrix metalloproteinases, including collagenases and other connective tissue-degrading enzymes [31]. Monocytes from diabetics have shown a hyperresponsive phenotype with overexpression of proinflammatory mediators such as interleukin-1*β*, tumor necrosis factor-*α*, and prostaglandin E_2_ [35, 36]. This exacerbation of the proinflammatory response in diabetics can lead to impaired wound healing and amplify connective tissues damage. This proinflammatory response may be further increased by the chemotactic properties of advanced glycation end-products for human monocytes which differentiate into the chronic inflammatory macrophages [32]. Degradation of newly synthesized collagen in connective tissues and alterations in the immune response can both contribute to progression of periodontal disease and impaired wound healing.

On the other hand, inflammation caused by inflammatory connective tissue disease such as periodontitis can trigger insulin resistance. Lipopolysaccharides from periopathogenic Gram-negative bacteria are able to induce tumor necrosis factor-alpha production by monocytes and macrophages. This cytokine can interfere with lipid metabolism, reduce glucose uptake by cells, and cause insulin resistance. An inflamed periodontium is highly vascular and may serve as a gate to the systemic circulation for bacterial products and produced local inflammatory mediators [37].

Periodontitis as an inflammatory condition leads to changes in cellular and humoral immunity. Polymorphonuclear cells and macrophages functions are affected; IL-2 and interferon gamma as well as cytokines important for humoral response such as IL-10 and transforming growth factor are produced. These changes in immune responses affect release of insulin and glycemic control [38, 39].

**Figure 2:**
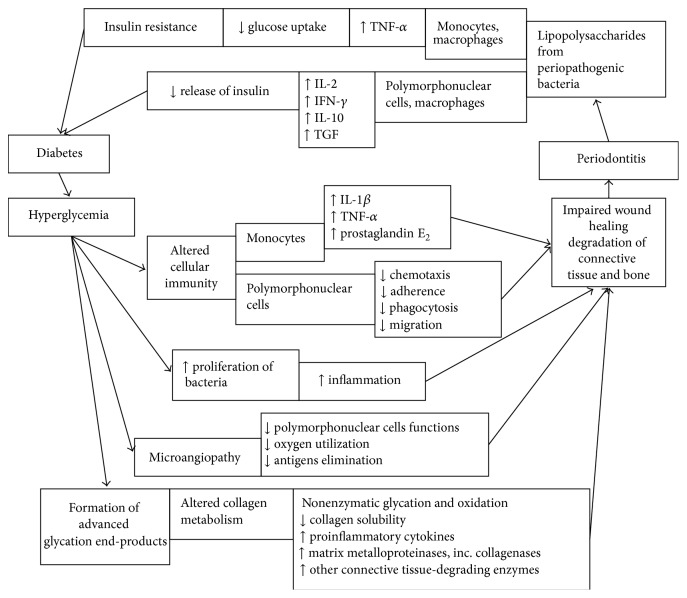
Associations between diabetes and periodontitis.

Periodontitis can thus obstruct glycemic control and obstructed glycemic control can further stimulate periodontal disease; a cycle worsening both conditions may be created (Figure 2).

Therefore, prevention and control of oral inflammatory diseases are essential for appropriate prevention and optimal management of diabetic complications [40].

### 2.3. Dental Caries

Regarding dental caries, the results of the studies are inconsistent. Dental caries is a multifactorial disease, and while some factors increase the risk of caries disease in type 1 diabetes, others reduce it.

Dental caries risk factors include oral cariogenic bacteria, intake of fermentable carbohydrates as a substrate for cariogenic bacteria, and sufficient time allowed for caries formation. The protective factors against caries include the saliva, oral hygiene, and fluorides [26]. The research has shown that the levels of cariogenic bacteria, particularly of* Streptococcus mutans*, are higher in diabetic patients and the proportion of individuals with high levels of cariogenic bacteria, particularly* Streptococcus mutans*, is higher in the diabetic population [26]. According to the nutritional recommendations, the diet of diabetic children should ideally be low in simple sugars, especially the so-called extrinsic sugars artificially added to food, while the so-called intrinsic sugars, contained mainly in fruits and vegetables, do not need to be restricted. The frequency of meals should be higher compared to healthy individuals; however, patient compliance is critical [41, 42]. The saliva of diabetics shows both quantitative and qualitative changes [43–48]. The oral hygiene habits and education of diabetic patients seem to be similar or slightly better compared to healthy individuals [18, 49].

There are studies that show a lower incidence of dental caries in diabetic children compared to the healthy peers, such as the one by Orbak et al., who also reported a higher prevalence of dental caries in permanent teeth of children with poorly controlled diabetes [14]. The extensive study by Lal et al. showed a lower prevalence of dental caries in deciduous teeth of diabetic children compared to the controls [50]. In the study by Siudikiene et al. performed in healthy and diabetic children aged 10–15 years, over a two-year follow-up, there were no significant differences in the incidence of dental caries between the two study groups [45]. The same pattern (i.e., nonsignificant differences in the dental caries incidence between healthy and diabetic children) was observed in a large trial by Lalla et al. [19] and in several recent studies from Brazil, Egypt, and Belgium, where the study groups consisted of approximately 50 diabetic children [51–53]. Contrary to the above findings, higher caries experience in diabetic children was observed by investigators in Kuwait, India, and Puerto Rico [54–56].

The importance of adequate metabolic control of diabetes was partially confirmed in the study by Siudikiene et al., which compared prevalence of dental caries and levels of mutant streptococci in pediatric diabetic patients with adequate and poor metabolic control of diabetes based on HbA1c levels [57]. A relationship between caries risk and metabolic control was found in Twetman et al.'s study as well [58].

Studies that assess caries experience using dmft/DMFT (decayed, missing, filled temporary/permanent teeth) index often find nonsignificant differences between healthy and diabetic subjects; however, individual components of the index reveal a higher proportion of dental caries (component d/D, decayed) in the control subjects and higher proportion of fillings (component f/F, filled) and teeth extracted due to caries (component m/M, missing) in the diabetic subjects [14, 59].

The results of the studies performed in adults with type 1 diabetes are also inconsistent regarding the prevalence of dental caries. Some studies report a higher incidence of cervical, interproximal, or root caries in diabetics. These findings are considered to be due to a higher content of glucose in the saliva and crevicular fluid in the diabetics [60].

### 2.4. Saliva

Compared to the healthy subjects, there are both quantitative and qualitative changes in the saliva of diabetic patients. Diabetics are considered to have a reduced salivary flow [26], especially that of unstimulated saliva. The study by Moreira observed that the unstimulated salivary secretion in children with diabetes versus the control group was 0.15 ± 0.1 mL/min and 0.36 ± 0.2 mL/min, respectively. As for the stimulated saliva, there were no significant differences observed [43]. Similar results were observed in the study by López et al., where the unstimulated salivary secretion in children with diabetes versus the control group was 0.15 ± 0.1 mL/min and 0.25 ± 0.1 mL/min, respectively [44]. The reduced unstimulated and stimulated salivary secretion in children with diabetes were demonstrated by Siudikiene et al. [45]. As for the qualitative parameters, the typical findings are lower buffering capacity and pH of the saliva in diabetics (salivary pH in pediatric patients with diabetes versus the control group was 6.0 ± 0.8 and 7.0 ± 0.6, resp.) [43], higher viscosity of the saliva, higher levels of carbohydrates, glucose, and total protein in the saliva [44, 45], and higher levels of IgA and IgG antibodies [45–47] but lower levels of antimicrobial proteins, for example, lactoferrin and lysozyme [48]. As for the calcium levels in the saliva of diabetic children, the results of the studies are inconsistent: there are reports of both higher calcium levels [43] (which results in an enhanced tartar build-up) and lower calcium levels [44].

Siudikiene et al. found an association between the incidence of dental caries and glucose levels in the saliva of diabetic children [45].

### 2.5. Oral Microflora

Differences in oral microflora of diabetics and healthy individuals may significantly influence the incidence of diseases caused by bacteria, such as periodontal impairment and dental caries.

It was observed that in diabetic individuals including adolescents there are differences in the type and amount of periodontal pathogens, their habitat, and patients' age of their occurrence compared to the healthy population [21, 22].

As for the levels of cariogenic species, the results of the studies are inconsistent. There are reports of both insignificant differences in the levels of* Streptococcus mutans* and lactobacilli in healthy and diabetic individuals [21, 45, 52] and higher proportion of diabetics with high levels of* Streptococcus mutans* in the saliva compared to the control group [26]. As for the levels of* Candida* species in the oral cavity, there are reports of not only no differences between the diabetic and healthy children [45, 52] but also higher levels in the diabetics versus the healthy population [26, 57].

With respect to inconsistent findings, more microbiological studies are needed to clarify potential differences between healthy and diabetic children.

### 2.6. Diet

A recommended diet for children with type 1 diabetes corresponds to traditional rules of rational nutrition. The intake of fat and in some cases also of proteins should be restricted, but according to the recent nutritional recommendations the intake of carbohydrates should be up to 50–60% of the daily caloric intake. Dietary carbohydrates should primarily come from the complex carbohydrates, starch and fiber, whereas foods and beverages high in simple carbohydrates, which result in a significantly increased postprandial glycemia, should be excluded [4].

The frequency of daily meals and snacks partially depends on whether the patient uses the insulin pen or pump and on the type of insulin regimen used. Nevertheless, the frequency of food intake in diabetics is generally higher compared to the healthy population, and a standard recommendation is six meals per day [4].

The nutritional compliance particularly in adolescent patients is debatable, and about half of the patients in this age group are considered to be noncompliant [4].

### 2.7. Metabolic Control of Diabetes

A long-term metabolic control of diabetes is usually verified based on not only glycated hemoglobin HbA1c levels but also the total daily dose of insulin and blood glucose fluctuations.

The metabolic control of diabetes is associated with the risk of both acute and chronic complications. The main chronic complications of type 1 diabetes include neuropathy and microangiopathy. In poorly controlled patients, there is a decreased leukocyte phagocytic activity but also antibody and cell-mediated immunity [4], which results in an increased risk of bacterial infections [4, 26].

The effect of proper metabolic control and occurrence of chronic complications of diabetes on the development of periodontal diseases has been confirmed by a number of studies [17, 22, 25], in contrast to dental caries. The study by Dusková and Broukal demonstrated a positive correlation between the levels of glycated hemoglobin (HbA1c) and those of* Streptococcus mutans* and* Candida* species in the oral cavity [22].

### 2.8. Behavioral Aspects

A positive correlation between the metabolic control of diabetes and the occurrence of oral pathologies may be related, besides the biological factors, to certain psychological features of the patients. These features influence the patient compliance and self-care related to both diabetes and oral health problems. This association was observed in a study performed in 149 insulin-dependent diabetics, which included a questionnaire survey, clinical assessment of oral health, and analysis of diabetic patients' medical records. The study showed that 82% of diabetic patients without gingivitis had a good metabolic control and lower mean levels of HbA1c versus the diabetics with gingivitis [61]. The patients with a higher self-reported frequency of tooth brushing and lower plaque index score had lower mean levels of HbA1c [62]. On the other hand, a positive correlation between an inadequate interdental plaque removal and noncompliance in diabetes self-care was found [61]. Although the abovementioned studies were performed in the adult populations, it can be assumed that the above findings can be applied also to the child and adolescent populations with diabetes.

## 3. Conclusion

The authors have used both latest and older studies to bring a comprehensive overview of the relationship between type 1 diabetes and oral health in children.

There have been a number of studies on this topic published in dentistry; however, unambiguous conclusions have been reached only concerning the relationship between diabetes and periodontal diseases. A number of studies have shown higher amounts of dental plaque and increased incidence of chronic gingivitis in both adults and children with type 1 diabetes. Periodontitis in children is rare both in healthy subjects and in children with type 1 diabetes. Yet some of the findings of well-performed studies indicate a higher risk of periodontitis in children with type 1 diabetes. Regarding the impact of diabetes on dental caries development, the results of clinical trials are inconsistent. However, it has been confirmed that some of minor caries risk factors are more or less prevalent in a diabetic population compared to a nondiabetic control group. Quantitative and qualitative salivary changes in diabetics have also been confirmed, even though particular detailed results of individual studies vary. Oral health studies focusing on behavioral features of diabetic patients yield even more interesting insights.

The latest studies tend to focus on the research of possible direct and indirect influence of type 1 diabetes on oral health and vice versa, but their results are not crystal clear in most aspects. On that account, relationships between diverse oral diseases and their causal factors and type 1 diabetes should become a subject of intensive research in the future.

## Figures and Tables

**Table 1:** Classification of diabetes by ADA [1].

I	Type 1 diabetes	Immune mediated
		Idiopathic

II	Type 2 diabetes	

III	Other specific types	

IV	Gestational diabetes mellitus	
